# Online pain neuroscience education and graded exposure to movement in breast cancer survivors: protocol of a randomized controlled trial

**DOI:** 10.3389/fmed.2024.1355964

**Published:** 2024-02-28

**Authors:** Patricia Martínez-Miranda, María Jesús Casuso-Holgado, Cristina García-Muñoz, María Jesús Muñoz-Fernández, José Jesús Jiménez-Rejano

**Affiliations:** ^1^Faculty of Nursing, Physiotherapy and Podiatry, Universidad de Sevilla, Seville, Spain; ^2^CTS 1110, UMSS Research Group, Andalusia, Spain; ^3^Instituto de Biomedicina de Sevilla, IBiS. Departamento de Fisioterapia, Universidad de Sevilla, Sevilla, Spain; ^4^Departamento de Ciencias de la Salud y Biomédicas, Universidad Loyola de Andalucía, Sevilla, Spain; ^5^Department of Physiotherapy, University School Francisco Maldonado, Osuna, Spain

**Keywords:** breast neoplasms, quality of life, pain neuroscience education, exercise therapy, yoga

## Abstract

**Introduction:**

Cancer-related chronic pain is an important sequelae that damages the quality of life of breast cancer survivors. Pain neuroscience education and graded exposure to movement are therapeutic tools that have been shown to be effective in the management of chronic pain in other populations. However, there are no previous studies that combine them after breast cancer.

**Objective:**

To evaluate the effectiveness of an online physiotherapy focused-person program which combines pain neuroscience education and graded exposure to movement for quality of life improvement in breast cancer survivors.

**Methodology:**

This protocol is a randomized controlled trial with a sample size of 40 breast cancer survivors with pain in the last 6 months. Participants will be allocated to the experimental or control group using a fixed size block randomization method. The evaluator and statistician will be blinded to participant allocation. Participants in the experimental group will receive a 12-week intervention based on pain neuroscience education and therapeutic yoga as a graded exposure to movement exercise; participants in the control group will continue with their usual cancer-related symptoms care. Both groups will receive an education booklet. The main outcome will be quality of life, measured by the Functional Assessment of Cancer Therapy – Breast (FACT–B+4); secondary, four outcomes related to pain experience (catastrophising, self-efficacy, kinesiophobia and fear-avoidance behaviors) will be also assessed. All variables will be assessed by two blinded evaluators at four timepoints. A mixed-model analyses of variance ANOVA (2 × 4) will be used to study the effects of the treatment on the dependent variables. All statistical tests will be performed considering a confidence interval of 95%. SPSS program will be used for the data analysis.

**Discussion:**

This research is expected to contribute to breast cancer rehabilitation field. The proposed intervention is also expected to improve self-care skills related to chronic pain and to empower women regarding the management of their symptoms and quality of life.

**Clinical trial registration:** https://clinicaltrials.gov/, NCT04965909.

## 1 Introduction

Currently, chronic pain is one of the sequalae with the highest incidence in breast cancer survivors, seriously impacting their quality of life and making it difficult for them to reintegrate into society and their workplace (1**–**3). According to a biopsychosocial perspective (4), the chronification of pain must be understood as a complex and multifactorial process involving biological, psychological, emotional and social factors (5).

Together with the advances in the understanding of chronic pain, several therapeutic approaches have emerged. Among them, interventions based on pain neuroscience education (PNE) and graded exposure to movement (GEM) have reported important benefits for different chronic pain conditions (6–17). PNE is defined as a therapeutic tool implemented by a healthcare professional aimed at the empowerment of people related to their pain process management (18–21), while GEM applies movement following the “Twin Peaks” metaphor proposed by Butler (22) to get more functionality associated with less painful experiences. In this clinical trial therapeutic yoga will be applied as a graded movement intervention in conjunction with techniques of movement representation (GEM-Y). Therapeutic yoga has demonstrated to be an effective exercise for the improvement of quality of life in adults with cancer (23), for addressing other adverse effects on breast cancer survivors (24), and to manage symptoms in other chronic painpopulations (14, 15). In addition, yoga is a body–mind exercise that allows us to follow biopsychosocial approach (5) and to adjust easily the intensity of exercise to each individual context. PNE has been scarcely investigated in breast cancer survivors (25, 26), and for our knowledge the combination of PNE with GEM-Y has never been studied in this population. As breast cancer prevalence and survivorship rate is growing exponentially in transitioned countries, but also cancer-related symptoms (27), it would be helpful to investigate biopsychosocial interventions aiming to improve quality of life in this population.

Thus, the purpose of this clinical trial will be to evaluate if an intervention combining PNE and GEM-Y is more effective than usual care for quality of life and chronic pain improvements in breast cancer survivors.

## 2 Methodology

### 2.1 Study design

A randomized controlled clinical trial will be carried out according to the Consolidated Standards of Reporting Trials (CONSORT) Statement (28). The Template for Intervention, Description and Replication Checklist (TIDieR) (29) will be used as a guide to provide transparency and make the intervention replicable. Also, the Standard Protocol Items: Recommendations for Interventional Trials (SPIRIT) was followed to develop this protocol. The protocol of this study has been registered on clinicaltrials.org with the registry number: NCT04965909.

### 2.2 Inclusion and exclusion criteria

Inclusion criteria: (1) Women aged between 18 and 65 years; (2) diagnosis of stage 0–III breast cancer; (3) primary treatment (surgery, radiotherapy and chemotherapy) completed at least 3 months ago but may still be receiving hormone therapy; (4) pain related to primary treatment in the last 6 months; (5) access to the Internet and an electronic device that allows the use of the applications used in this study and skills for their use or assistance from a close person who has them; (6) ability to communicate fluently verbally and in writing in the language of the research team (Spanish); and (7) approval to participate in the study by the coordinator of the health team that assisted during the course of cancer and its treatment.

Exclusion criteria: (1) another previous type of cancer or breast cancer recurrence in a period of less than 1 year; (2) medical diagnosis of a neurological or autoimmune disease that limits or prevents exercise; (3) some type of pathology that is associated with a contraindication to physical exercise; and (4) the diagnosis of serious psychiatric or neurologic disorders that do not allow the participant to follow orders.

### 2.3 Sampling method and sample’s size calculation

**Figure 1:**
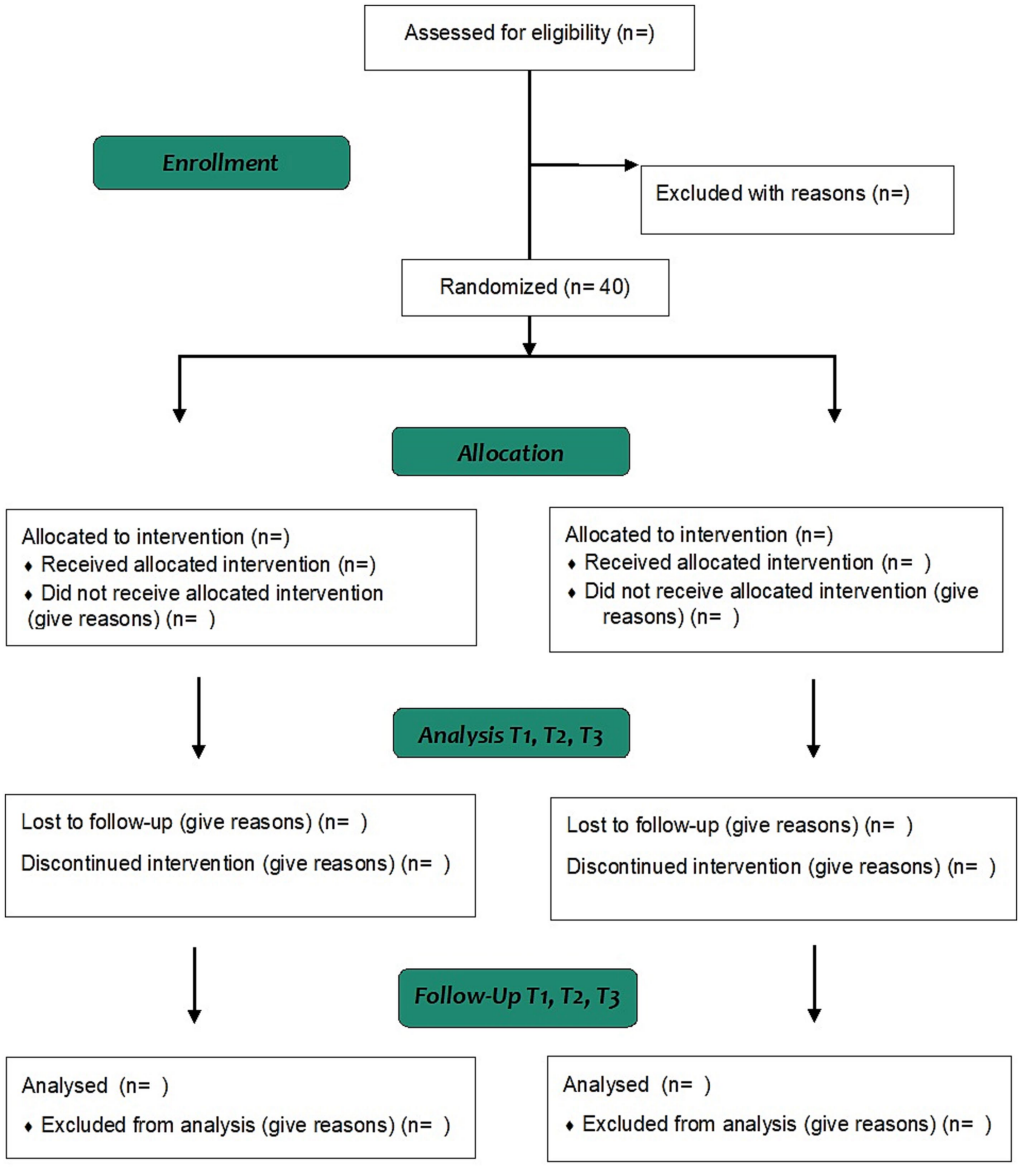
CONSORT flow diagram.

For sampling, non-probabilistic convenience and snowball methods will be used. The sample size was calculated based on the change in FACT-B score between the treatment and control groups at week 12. According to a previous study (30), it is estimated that patients in the intervention group would have a difference in FACT-B score of 10.11 points or more compared to the control group (*F*-value = 4.86). This difference is above the minimal important difference reported for this measure (7–8 points) (31) and results in an expected partial Eta2 effect size of 0.049. Considering 2 groups, 4 measurements, a type I risk or α 0.05, type II risk or β 0.20 (study power of 80%) and an estimated dropout rate of 15%, a total of 40 participants (20 per group) are needed to be enrolled. Sample size was calculated using the G*Power software, version 3.1.9.7 (Heinrich-Heine University, Düsseldorf, Germany). Figure 1 shows the flow chart of the study.

### 2.4 Subjects’ recruitment

The sample for this study will be recruited through the dissemination of the project using social networks and with the collaboration of three Spanish breast cancer survivor support associations (Amama Sevilla, AGAMAMA and ASAMMA). Participation in the study will be voluntary. All participants will be facilitated by written informed consent that must be signed to be part of the clinical trial.

### 2.5 Group assignment and masking

This study will have two groups (experimental and control). For assignment, a random method will be carried out using an online tool called ‘random allocation software’ (2.0 version). A stratified allocation will be applied according to the women’s age (≤45 years old or >45 years old). On each of the strata, a randomization will be carried out by blocks of constant size. The assignment sequence will be hidden from the evaluator and the study subjects through an automated assignment system. The preparation of the sequence, the inclusion of the individuals in each group and the assignment of the treatments will be carried out by different members of the research team. On the other hand, the evaluator and statistician will be blinded. Nonetheless, the therapist and subject will not be able to be blinded because of the type of intervention.

### 2.6 Outcomes and data collection

The main outcome of this trial is quality of life related to health, measured by the Functional Assessment of Cancer Therapy – Breast (FACT–B+4) (32); five secondary outcomes related to chronic pain experiences will be also measured: intensity of pain, catastrophising level, pain self-efficacy, kinesiophobia, and fear-avoidance behaviors.

#### 2.6.1 The Functional Assessment of Cancer Therapy – Breast+4

The Functional Assessment of Cancer Therapy – Breast (FACT–B+4) (32) is a 41-item instrument designed to measure six domains of quality of life in patients with breast cancer: physical (PWB), social (SWB), emotional (EWB) and functional (FWB), breast cancer subscale (BCS) and lymphedema subscale (ARM). The overall score of the FACT–B+4 ranges from 0 to 148 points (obtained from the sum of the PWB, SWB, FWB and BCS). The score of the PWB, SWB, EWB and FWB ranges between 0 and 28 points, the score of BCS between 0 and 40 points and the score of ARM between 0 and 20 points. In all of them, a higher score translates to a better quality of life. The alpha coefficient (internal consistency) and test–retest reliability for the FACT–B+4 overall score was high (alpha = 0.87; intraclass correlation coefficient: 0.986). This measure has been widely used in breast cancer population previously (33).

#### 2.6.2 Brief Pain Inventory – Short Form

The Modified Brief Pain Inventory – Short Form (BPI–SF) (34) is a 9-item instrument designed to measure pain intensity and pain interference with the daily activities, which has been previously assessed in breast cancer population for this purpose (25, 26). The questionnaire has two subareas, one related to pain intensity, whose score ranges from 0 to 50, with a higher score being an indication of greater intensity; and another related to the interference of pain in activities of daily living, whose score ranges from 0 to 70, with a higher score being indicative of a greater impact on daily life. The internal consistency and the test–retest reliability between dimensions were good (0.87 and 0.89) and low to moderate (0.53 and 0.77), respectively.

#### 2.6.3 Pain Catastrophizing Scale

Pain Catastrophizing Scale (PCS) (35) is one of the most widely used instruments to assess the degree of catastrophizing of pain as a result of various pathologies or diseases, including breast cancer population (25, 36). The scale consists of 3 subscales (rumination, magnification and helplessness), whose items will be valued from 0 (nothing) to 4 (all the time) to obtain a total score that ranges from 0 to 52. A higher score translates into a higher level of catastrophizing. The scale has adequate internal consistency (Cronbach’s alpha = 0.79), test–retest reliability (intraclass correlation coefficient = 0.84) and sensitivity to change (effect size ≥2).

#### 2.6.4 Pain Self-Efficacy Questionnaire

The Pain Self-Efficacy Questionnaire (PSEQ) (37) is a 22-item instrument designed to measure self-efficacy level related to pain. Each item is scored from 0 to 10. Here, 0 is equal to ‘I think I am totally incapable’ and 10 is equal to ‘I think I am totally capable’. The total score ranges from 0 to 220. A higher score on the questionnaire corresponds to a higher level of self-efficacy. The internal consistency and test–retest reliability between dimensions were 0.91 and 0.75, respectively. This measure has been previously used in cancer survivors with pain (38, 39).

#### 2.6.5 Tampa Scale for Kinesiophobia-11

Tampa Scale for Kinesiophobia (TSK-11) (40) is one of the most commonly used to evaluate kinesiophobia in patients with pain, including breast cancer population (36). It is composed of two factors (avoidance of activity and harm) with a total of 11 items that are valued from 1 (totally disagree) to 4 (totally agree). The total score obtained ranges from 11 to 44. More punctuation shows a higher kinesiophobia level. The internal consistency (Cronbach’s alpha = 0.79) found for this scale is good.

#### 2.6.6 Fear Avoidance Components Scale Questionnaire – Spanish Version

The Fear Avoidance Components Scale Questionnaire – Spanish Version (FACS – SP) (41) is a questionnaire that allows us to evaluate a patient’s fear of pain and consequent avoidance of physical activity due to fear. The questionnaire consists of 20 items in which a patient rates his agreement with each statement on a 6-point Likert scale. Where 0 = completely disagree and 6 = completely agree. There is a maximum score of 100. A higher score indicates more strongly held fear-avoidance beliefs. Five severity levels are available for clinical interpretation: subclinical (0–20), mild (21–40), moderate (41–60), severe (61–80) and extreme (81–100). It has been previously used in breast cancer population (42).

**Figure 2:**
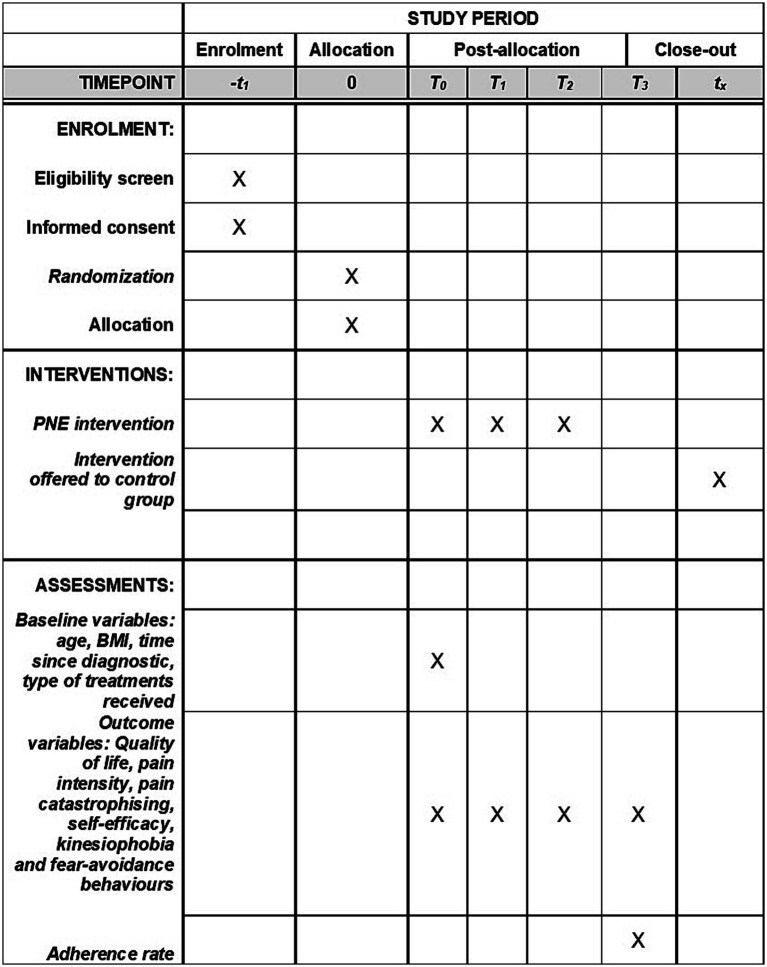
SPIRIT schedule.

All outcomes will be assessed at four different timepoints: before intervention (T0), after four-week PNE (T1), after 12-week complete intervention PNE + GEM-Y (T2) and after 3-month of follow-up (T3) (Figure 2). The outcomes will be assessed using the aforementioned validated scales or questionnaires that women will complete by themselves. Moreover, extra information related to their pain context will be collected by two trained evaluators in an online meeting. All data will be collected on a standardized sheet. It will be encrypted and only members of the research team will have access.

### 2.7 Description of the intervention in the experimental and control group

**Figure 3:**
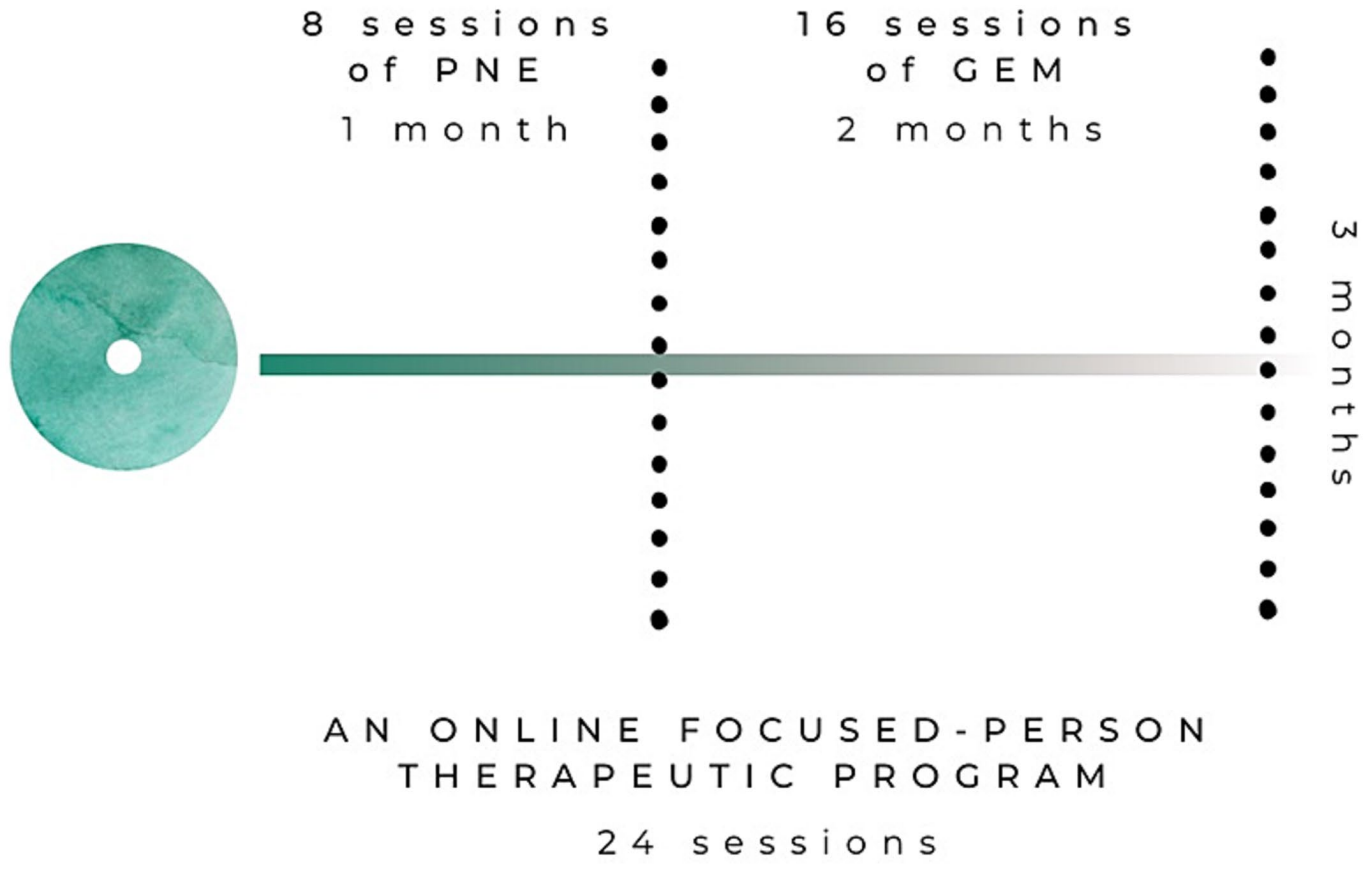
Overview of the experimental intervention.

An online focused-person therapeutic program, a more up-to-day modality for this type interventions (43), that combines PNE and GEM-Y will be implemented in the experimental group. The sessions will be applied in groups of 10–15 participants, with 3 months being the duration of the entire program. The program will have two parts: the first will involve 8 sessions of PNE during the first month (2 sessions each week, 1 h/session), while the second will involve the use of 16 sessions of GEM-Y during the following 2 months (2 sessions each week, 1 h/session). Figure 3 shows an overview of the experimental intervention.

**Figure 4:**
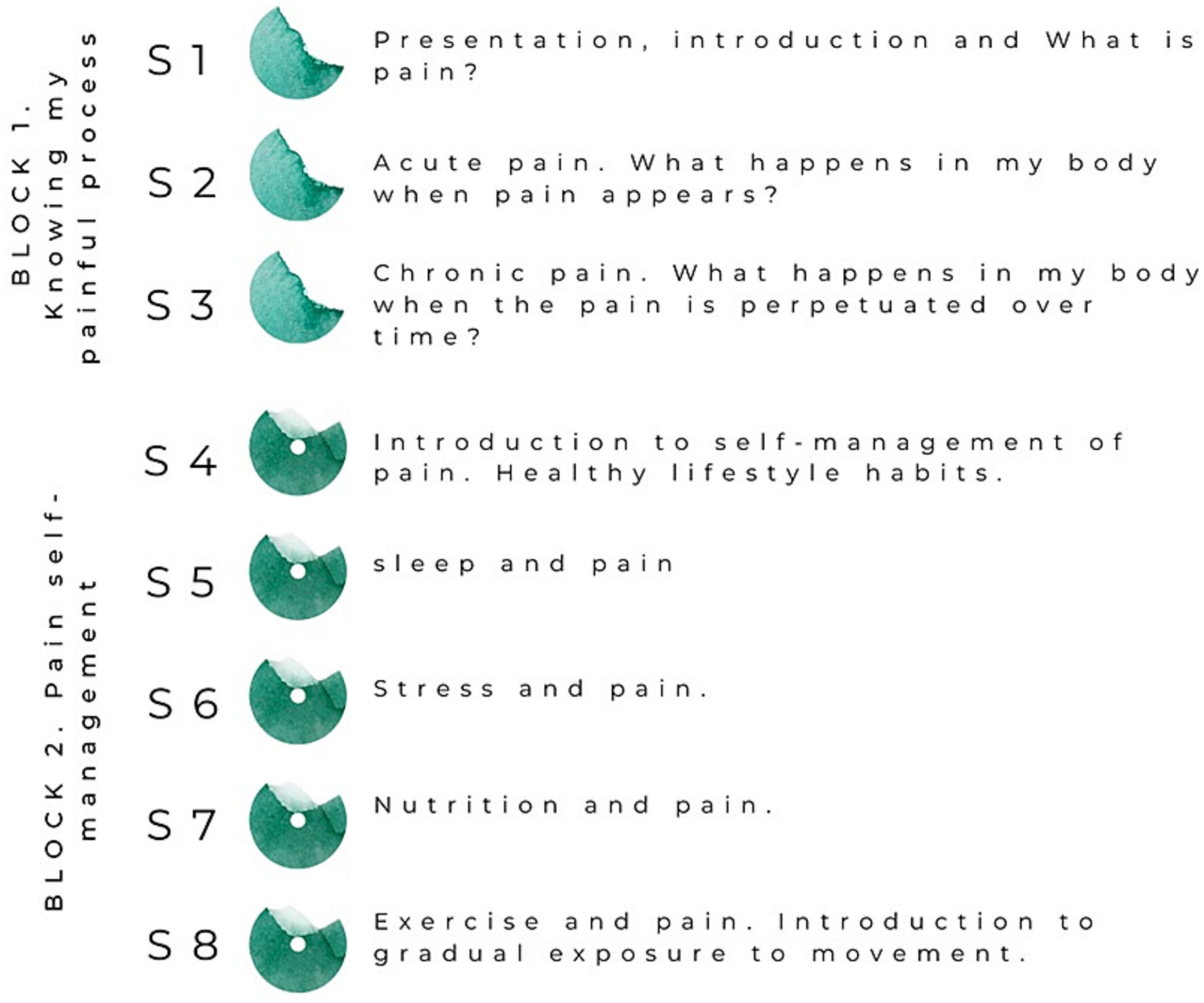
Overview of the PNE sessions.

PNE sessions are divided into two content blocks: Block 1. *Knowing my painful process* and Block 2. *Pain self-management.* The Block 1 is divided into three sessions with the following educational topics: sessions (1–3) the concept of pain, acute pain and chronic pain, respectively; session (4) the concept of self-management in relation to pain and healthy habits; sessions (5–8) sleep, stress, diet and exercise habits in relation to chronic pain, respectively. A brief theoretical introduction to the GEM will be also given in the last session. A more detailed description of the content of all PNE sessions and the educational strategies to be followed is presented in the Supplementary material. Figure 4 shows an overview of the PNE programme.

GEM-Y sessions will be organised into four phases: theoretical content of the session, ‘pranayama’ or breathing exercises, ‘dhyana’ or guided meditation, and ‘asanas’ or postures and movements. Moreover, each session will focus on a different part of the body. In this way, yoga is used as a method of therapeutic exercise together with movement representation techniques (44, 45). The program will be delivered by a trained therapist following the principles of progression, gradualness and individualisation proposed by “Twin Peaks” metaphor (22). Thus, at the beginning of the sessions each participant must identify their pain baseline in order to apply an optimal dose of exercise (principle of individualisation). Instructions are given to the participants so that they can always adapt the level of effort to their needs and their progression (more functionality with less associated pain). With regard to the principles of progression and gradualness, since each participant will have a different starting point at the beginning of the programme and will move to their own level, the rate of progression of each participant must also be different. The intensity of the proposed exercises will be progressively adapted to the needs of the group by varying the following parameters: complexity of the ‘asanas’, volume of work (number of exercise blocks and repetitions), control of the relationship work time - rest time ratio. To identify the needs and progress of the group we will use the feedback collected from the participants at the end of each session, and the weekly pain diaries. A more detailed description of the GEM-Y sessions can be found in the Supplementary material.

The whole intervention will be implemented online using the videoconference platform of the University of Seville. Furthermore, WhatsApp and e-mail will be used during the study to give information, provide material or answer queries. Participants’ attendance will be recorded, along with the reasons for non-attendance.

Participants in the control group will only receive traditional biomedical information (26, 36), i.e., explanations of perceived pain based on tissue issues, and general oncological recommendations for analgesia. They will not receive any additional educational or movement-based intervention during the study period. They will be offered the content of the program after the follow-up period for ethical reasons. An online educational booklet will be provide to both groups.

### 2.8 Method for data analysis

The statistical processing of the data will be conducted with the PASW Advanced Statistics, version 26.0 (IBM Corp, New York, NY, United States). Intention-to-treat principles will be considered for all analyses. The normal distribution of the variables will be assessed with the Shapiro–Wilk test. Descriptive data will be reported as mean (standard deviation), median (interquartile range Q_3_–Q_1_) or in percentages. Baseline homogeneity will be tested with Chi-square or Fisher’s exact tests; student’s t test or Mann–Whitney *U*-test.

In those variables in which the 4 measurements are adjusted to normality in both groups, a mixed-model analyses of variance ANOVA (2 × 4) will be used to differences in the outcomes after intervention, with group (PNE + GEM-Y or control) as a between-subject factor and time (the different measurements performed) as a within-subject factor. The hypothesis of interest will be the interaction group by time with an *a priori* alpha level of 0.05. Partial eta squared (η2) will be calculated to estimate the effect size. If any of the measurements do not adjust to normality, we will use the Friedman ANOVA test and the effect size will be calculated as Rosenthal’s r with the formula: *r* = Z/√N. All statistical tests will be performed considering a confidence interval of 95%.

### 2.9 Ethical considerations

This protocol has the approval of the Andalusian Research Ethics Committee (CEI) of the Virgen Macarena - Virgen del Rocío University Hospitals, Sevilla, Spain (protocol code: 2170-N-20; date of approval: 14th June 2021). This clinical trial will follow the recommendations of the Declaration of Helsinki (46) and Spanish legal regulation regarding clinical research in humans (Law 14/2007 on Biomedical Research) (47).

Participants will be verbally informed in a clear and precise way of all aspects of the study. Written information, informed consent and revocation sheet will be given to all participants. Informed consent will be signed before randomization process. All data will be managed in accordance with Spanish Law 3/2018 on the Protection of Personal Data and Guarantee of Digital Rights (48).

## 3 Reflexive discussion

The current trial aims to determine if the application of an online programme combining PNE with GEM-Y presents higher efficacy than no intervention in improving quality of life and chronic pain in breast cancer survivors. Preliminary evidence has showed that PNE may reduce pain intensity and pain catastrophizing in cancer survivors with persistent pain, but no effect on quality of life was observed (49). Particularly for breast cancer, previous findings about the effect of PNE are controversial. Cramer et al. (24) evaluated the effect of perioperative PNE on pain chronification 1 year after surgery and reported that PNE was more beneficial than general biomedical information for this purpose. In contrast, Manfuku et al. (25) concluded that perioperative PNE had not significant effect on pain-related disability or pain intensity 18 months after surgery.

Yoga has been showed to be an effective exercise modality for improving overall quality of life in people with cancer (50). In breast cancer in particular, the majority of studies to date support this benefit (24, 51–53), but no effects have been reported in other cases (54, 55). The effects of yoga on cancer-related pain have been scarcely investigated, with controversial findings for pain severity reduction in breast cancer population (51, 56).

Although the evidence for PNE in breast cancer is still limited, we consider it is possible that the combination of this intervention with yoga may benefit the quality of life and chronic pain experience of breast cancer survivors. To our knowledge, this type of programme has not been previously tested and fits with future directions for pain management in cancer survivors (57). Thus, this clinical trial is an innovative proposal that could have significant benefits for women’s health and their resources for coping with chronic pain. In addition, women and health policies could benefit from a reduction in medication use and socio-economic savings. In addition, the results of this trial will be disseminated in peer-reviewed journals and at international conferences, and shared with participants and other people with cancer.

Finally, some limitations need to be discussed. First, the follow-up period could be considered short as most of the educational interventions in this population consider longer periods; however, when educational interventions are presented in an online modality, it is common to consider this time period (43). Secondly, the proposed snowball sampling method could limit the generalisability of our results, as well as the representativeness of the subjects analyzed.

## Ethics statement

The studies involving humans were approved by Andalusian Research Ethics Committee (CEI) of the Virgen Macarena - Virgen del Rocío University Hospitals, Sevilla, Spain (protocol code: 2170-N-20; date of approval: 14th June 2021). The studies were conducted in accordance with the local legislation and institutional requirements. The participants provided their written informed consent to participate in this study.

## Author contributions

PM-M: Conceptualization, Methodology, Writing – original draft, Writing – review & editing. MC-H: Conceptualization, Funding acquisition, Methodology, Project administration, Supervision, Writing – original draft, Writing – review & editing. CG-M: Writing – original draft, Writing – review & editing. MM-F: Writing – original draft, Writing – review & editing. JJ-R: Conceptualization, Software, Supervision, Writing – original draft, Writing – review & editing.
