# Supplementary material for: Online pain neuroscience education and graded exposure to movement in breast cancer survivors: protocol of a randomized controlled trial
Source: Front Med (Lausanne). 2024 Feb 28;11:1355964. doi: 10.3389/fmed.2024.1355964 (PMC10933039; doi:10.3389/fmed.2024.1355964)
Supplement: Supplementary file 1 [file Data_Sheet_1.docx]

Supplementary Material

Description of the intervention based on TIDieR checklist

Program name: An online focused-person therapeutic program based on Pain Neuroscience Education (PNE) combines with Graded Exposure to Movement based intervention throughout therapeutic yoga (GEM-Y).

Brief description and WHY: This program aims to improve the quality of life and pain in breast cancer survivors. For this purpose, combines two tools (PNE and GEM) that have shown efficacy in other populations with chronic pain^1-12^. This program is created based on the biopsychosocial^13^ model and the focused-person^14^ attendance and tries to empower women regard to their pain management and their health. The program will be applied in a group format.

WHO: This program will be implemented by a trained physiotherapist according to the established plan.

WHERE: This is an online program, so the sessions will be implemented using a videoconferences platform (Blackboard Learn by University of Seville). It will also be used other online resources to give information, solve doubts and coordinate the group such as a WhatsApp group or e-mail, for instance.

HOW/Organisation: This 3-months program have two parts. First of them apply 8 sessions of PNE during the first month while in the second one 16 sessions of GEM-Y during the following two months. There will be two sessions weekly in all the program and each session will have a duration of one hour. In total, 24 therapeutic sessions will be implemented (Figure S1). All the sessions will be applied in group format (10 – 15 participants). The sessions schedule will be agreed between the participants group and the physiotherapist.

# CONTENT OF THE INTERVENTION:

*Block 1. Pain Neuroscience Education (PNE)*

This is a therapeutic tool implemented by a health professional that aimed at empowering people in relation to their pain process management. PNE promote the metacognition and self-knowledge of the person of their own history of pain, the identification of the key factors that are influencing the perpetuation of their pain in each pain history, the re-education of maladaptive beliefs and thoughts, the reduction of uncertainty, fear and the maladaptive emotional response to pain, the change of maladaptive behaviours, coping strategies and habits, and the definition of SMART objectives and a focused-person plan of detailed intervention^15-18^.

PNE will be implemented following the 'Meaningful Learning Theory'^19^, so the motivation of the participants and an active coping will be essential throughout the course of the program.^20^ Furthermore, the words and expressions used by the physiotherapist will be used with caution, trying to avoid any nocebo effect or suppression of thought related to pain and favouring placebo^21^.

Eight online sessions of PNE will be implemented during the first month of the program (2 weekly; 1 hour/session). The content of PNE could be divided into two blocks: Block 1, knowing my painful process and Block 2, pain self-management. The content of Block 1, knowing my painful process is: S1) In this session there will be a presentation of the program, the therapeutic team, and the participants, as well as a brief introduction to it, delving into the concept of pain. This session will be very focused on starting a good therapeutic alliance; S2) in the second session, acute pain and its biological meaning will be discussed, focusing the session on helping participants to know what happens in their body when pain begins, and to break myths about it; S3) in the last session of this block we will focus on chronic pain. The session will be aimed at helping participants understand what happens in their body when pain is perpetuated over time, as well as those key concepts whose understanding is a basic pillar for the self-management tools proposed by the program. The content of Block 2, pain self-management is: S4) There will be an introduction to the self-management tools that support our therapeutic program, transferring its focus to the participants. An introduction will be made to the importance of healthy lifestyle habits in relation to the management of chronic pain. S5-S8) during the following sessions, each of the pillars that are identified as important in the pain self-management approach proposed by our program will be specifically addressed. In each session, the relationship between the lifestyle in question and chronic pain will be shown to the participants, and clues or proposals will be given to create more adaptive behaviours with respect to it. In S8, a brief theoretical introduction to the GEM will also be given. Some of the most important points that will be consider during PNE will be the creation of a therapeutic alliance^14^, the group work, the focused-person^14^ and biopsychological approach^13^ and the search of therapeutic goals that will be consensus between person-therapist.

During the sessions the material used will be online presentations and audio-visual educative material. The therapist will provide information and order concepts based on questions to the group, debates, and group dynamics, and build learning based on it. At the end of each session, the participants will be provided with support material that synthesizes the key ideas of each session and proposals for activities to do at home. Additionally, a weekly online pain diary will be provided to work at home. Said diary is an individualized questionnaire aimed at motivating personal work and active coping and will be made up of 3 sections: the first will be aimed at reinforcing the key learning from the week's sessions, the second at stimulating individual self-knowledge of each painful experience and the context that surrounds it, as well as the woman's expression, and the third one to a monitoring of pain and visual reinforcement to test the possible advances during the program.

***Block 2. Graded Exposure to Movement based intervention throughout therapeutic yoga (GEM-Y)***

The structure of each session will be organised in four blocks: theoretical content of the session, 'pranayama' or breathing exercises, 'dhyana' or guided meditation, and 'asanas' or postures and movements. Thus, yoga will be used as a method of therapeutic exercise together with movement representation techniques^23-24^.

GEM-Y will be applied following the principles of progression, gradualness and individualization proposed by “Twin Peaks” metaphor.^22^ According to this model,^22^ to carry out an adequate gradual exposure to movement that allows our nervous system and other systems to adapt to the given stimuli, the main pillar of each session is the perception of the baseline of pain of each participant. The identification of this baseline will allow us to apply an optimal dose of exercise in each session and for each participant (principle of individualization). For this principle, Borg Scale will be also used to recognize each perceived effort in the theoretical content of each session, as well as in the last PNE session. For this purpose, instructions, modifications and resources will be given to the participants so that they are able to always adapt the level of effort to their needs, and an individual progression (more functionality with less associated pain) will be sought between the first and last session of gradual exposure to movement.

Regarding the principles of progression and gradualness, since the baseline of each participant will be different at the beginning of the program and will move to an own step, the rate of progression of each participant must also be different. The intensity of the proposed exercises will progress, adapting to the needs of the group by varying the following parameters: complexity of the 'asanas', volume of work (number of exercise blocks and repetitions), control of the relationship work time - rest time. To identify the needs and progression of the group we will use the feedback collected by the participants at the end of each session, and the weekly pain diaries. Each session will be centred into a different body zone.

Our exercise program is designed to be a flexible and adaptable program to the specific needs of each group and each woman. Thus, the search for the optimal dose of exercise will constitute the basis of each session, the program being the one that adapts to the gradual progression of the participant, and not the participant at the pace established by a rigid program.

The material necessary to do the sessions will be comfortable clothes, a mat, a block, a cushion, and a blanket. Participants will be asked to try to do the sessions in a quiet place. During this second part of the study, pain diaries will also be used as a supplementary method of work at home. In this case, it will be aimed at each participant expressing their individual weekly experience, testing progress and being able to make a good adjustment of the dose of exercise applied.

References

1. Watson JA, Ryan CG, Cooper L, Ellington D, Whittle R, Lavender M, et al. Pain Neuroscience Education for Adults With Chronic Musculoskeletal Pain: A Mixed-Methods Systematic Review and Meta-Analysis. J Pain. 2019 Oct;20(10):1140.e1-1140.e22.
2. Siddall B, Ram A, Jones MD, Booth J, Perriman D, Summers SJ. Short-term impact of combining pain neuroscience education with exercise for chronic musculoskeletal pain: a systematic review and meta-analysis. Pain. 2022;163(1):e20-e30. doi:10.1097/j.pain.0000000000002308
3. Saracoglu I, Akin E, Aydin Dincer GB. Efficacy of adding pain neuroscience education to a multimodal treatment in fibromyalgia: A systematic review and meta‐analysis. Int J Rheum Dis. 2022;25(4):394-404. doi:10.1111/1756-185X.14293
4. Bonatesta L, Ruiz-Cárdenas JD, Fernández-Azorín L, Rodríguez-Juan JJ. Pain Science Education ^Plus^ Exercise Therapy in Chronic Nonspecific Spinal Pain: A Systematic Review and Meta-analyses of Randomized Clinical Trials. J Pain. 2022;23(4):535-546. doi:10.1016/j.jpain.2021.09.006
5. Ordoñez-Mora LT, Morales-Osorio MA, Rosero ID. Effectiveness of Interventions Based on Pain Neuroscience Education on Pain and Psychosocial Variables for Osteoarthritis: A Systematic Review. Int J Environ Res Public Health. 2022;19(5):2559. doi:10.3390/ijerph19052559
6. De Jong JR, Vlaeyen JW, Onghena P, Goossens ME, Geilen M, Mulder H. Fear of movement/(re)injury in chronic low back pain: education or exposure in vivo as mediator to fear reduction? Clin J Pain. 2005 Jan-Feb;21(1):9-17; discussion 69-72. doi: 10.1097/00002508-200501000-00002. PMID: 15599127.
7. De Jong JR, Vlaeyen JWS, Onghena P, Cuypers C, den Hollander M, Ruijgrok J. Reduction of pain-related fear in complex regional pain syndrome type I: the application of graded exposure in vivo. Pain. 2005 Aug;116(3):264-275. doi: 10.1016/j.pain.2005.04.019. PMID: 15964686.
8. Woods MP, Asmundson GJG. Evaluating the efficacy of graded in vivo exposure for the treatment of fear in patients with chronic back pain: a randomized controlled clinical trial. Pain. 2008 Jun;136(3):271-280. doi: 10.1016/j.pain.2007.06.037. Epub 2007 Aug 22. PMID: 17716819.
9. Zhu F, Zhang M, Wang D, Hong Q, Zeng C, Chen W. Yoga compared to non-exercise or physical therapy exercise on pain, disability, and quality of life for patients with chronic low back pain: A systematic review and meta-analysis of randomized controlled trials. PLoS One. 2020 Sep 1;15(9):e0238544. doi: 10.1371/journal.pone.0238544. PMID: 32870936; PMCID: PMC7462307.
10. Lauche R, Hunter DJ, Adams J, Cramer H. Yoga for Osteoarthritis: a Systematic Review and Meta-analysis. Curr Rheumatol Rep. 2019 Jul 23;21(9):47. doi: 10.1007/s11926-019-0846-5. PMID: 31338685.
11. Bravo C, Skjaerven LH, Guitard Sein-Echaluce L, Catalan-Matamoros D. Effectiveness of movement and body awareness therapies in patients with fibromyalgia: a systematic review and meta-analysis. Eur J Phys Rehabil Med. 2019 Oct;55(5):646-657. doi: 10.23736/S1973-9087.19.05291-2. Epub 2019 May 15. PMID: 31106558.
12. Danon N, Al-Gobari M, Burnand B, Rodondi PY. Are mind-body therapies effective for relieving cancer-related pain in adults? A systematic review and meta-analysis. Psychooncology. 2022 Mar;31(3):345-371. doi: 10.1002/pon.5821. Epub 2021 Sep 21. PMID: 34545984; PMCID: PMC9291932.
13. Gatchel RJ, Peng YB, Peters ML, Fuchs PN, Turk DC. The biopsychosocial approach to chronic pain: Scientific advances and future directions. Psychol Bull [Internet]. 2007;133(4):581–624. Available from: <http://doi.apa.org/getdoi.cfm?doi=10.1037/0033-2909.133.4.581>
14. Martinez-Calderon J, Flores-Cortes M, Morales-Asencio JM, Luque-Suarez A. Which Psychological Factors Are Involved in the Onset and/or Persistence of Musculoskeletal Pain? An Umbrella Review of Systematic Reviews and Meta-Analyses of Prospective Cohort Studies. Clin J Pain [Internet]. 2020 Aug;36(8):626–37.
15. (WHO), W. H. O. R. O. for the E. M. (2012). Health education: theoretical concepts, effective strategies and core competencies. Regional Office for the Eastern Mediterranean, Cairo.
16. Przybylska, D., Borzęcki, A., Drop, B., Przybylski, P., & Drop, K. (2014). Health Education as an Important Tool in the Healthcare System. Polish Journal of Public Health, 124(3), 145–147. <https://doi.org/10.2478/pjph-2014-0032>
17. Anderson, L., Brown, J. P., Clark, A. M., Dalal, H., Rossau, H. K. K., Bridges, C., & Taylor, R. S. (2017). Patient education in the management of coronary heart disease. Cochrane Database of Systematic Reviews. <https://doi.org/10.1002/14651858.CD008895.pub3>
18. Maunsell, E., Lauzier, S., Brunet, J., Pelletier, S., Osborne, R. H., & Campbell, H. S. (2014). Health-related empowerment in cancer: Validity of scales from the Health Education Impact Questionnaire. Cancer, 120(20), 3228–3236. <https://doi.org/10.1002/cncr.28847>
19. Vervoort T, Trost Z. Examining Affective-Motivational Dynamics and Behavioral Implications Within The Interpersonal Context of Pain. J Pain [Internet]. 2017 Oct;18(10):1174–83. Available from: <https://linkinghub.elsevier.com/retrieve/pii/S1526590017305345>
20. Taccolini Manzoni AC, Bastos de Oliveira NT, Nunes Cabral CM, Aquaroni Ricci N. The role of the therapeutic alliance on pain relief in musculoskeletal rehabilitation: A systematic review. Physiother Theory Pract [Internet]. 2018 Dec 2;34(12):901–15. Available from: <https://www.tandfonline.com/doi/full/10.1080/09593985.2018.1431343>
21. Rossettini G, Carlino E, Testa M. Clinical relevance of contextual factors as triggers of placebo and nocebo effects in musculoskeletal pain. BMC Musculoskelet Disord [Internet]. 2018 Dec 22;19(1):27. Available from: <https://bmcmusculoskeletdisord.biomedcentral.com/articles/10.1186/s12891-018-1943-8>
22. Butler DS. Explicando el dolor. 2a edición. Moseley L, Torres Cueco R, editors. Adelaide: Noigroup Publications; 2013.
23. Thieme H, Morkisch N, Rietz C, Dohle C, Borgetto B. The Efficacy of Movement Representation Techniques for Treatment of Limb Pain--A Systematic Review and Meta-Analysis. J Pain. 2016 Feb;17(2):167-80. doi: 10.1016/j.jpain.2015.10.015. Epub 2015 Nov 6. PMID: 26552501.
24. Bowering KJ, O'Connell NE, Tabor A, Catley MJ, Leake HB, Moseley GL, Stanton TR. The effects of graded motor imagery and its components on chronic pain: a systematic review and meta-analysis. J Pain. 2013 Jan;14(1):3-13. doi: 10.1016/j.jpain.2012.09.007. Epub 2012 Nov 15. PMID: 23158879.
